# Akt attenuates apoptotic death through phosphorylation of H2A under hydrogen peroxide-induced oxidative stress in PC12 cells and hippocampal neurons

**DOI:** 10.1038/srep21857

**Published:** 2016-02-22

**Authors:** Ji Hye Park, Chung Kwon Kim, Sang Bae Lee, Kyung-Hoon Lee, Sung-Woo Cho, Jee-Yin Ahn

**Affiliations:** 1 Department of Molecular Cell Biology, Samsung Biomedical Research Institute, Sungkyunkwan University School of Medicine, Suwon 440-746, Korea; 2 Department of Anatomy and Cell Biology, Samsung Biomedical Research Institute, Sungkyunkwan University School of Medicine, Suwon 440-746, Korea; 3 Center for Molecular Medicine, Samsung Biomedical Research Institute, Sungkyunkwan University School of Medicine, Suwon 440-746, Korea; 4 Department of Biochemistry and Molecular Biology, University of Ulsan, College of Medicine, Seoul 138-736, Korea

## Abstract

Although the essential role of protein kinase B (PKB)/Akt in cell survival signaling has been clearly established, the mechanism by which Akt mediates the cellular response to hydrogen peroxide (H_2_O_2_)-induced oxidative stress remains unclear. We demonstrated that Akt attenuated neuronal apoptosis through direct association with histone 2A (H2A) and phosphorylation of H2A at threonine 17. At early time points during H_2_O_2_ exposure of PC12 cells and primary hippocampal neurons, when the cells can tolerate the level of DNA damage, Akt was activated and phosphorylated H2A, leading to inhibition of apoptotic death. At later time points, Akt delivered the NAD^+^-dependent protein deacetylase Sirtuin 2 (Sirt 2) to the vicinity of phosphorylated H2A in response to irreversible DNA damage, thereby inducing H2A deacetylation and subsequently leading to apoptotic death. Ectopically expressed T17A-substituted H2A minimally interacted with Akt and failed to prevent apoptosis under oxidative stress. Thus Akt-mediated H2A phosphorylation has an anti-apoptotic function in conditions of H_2_O_2_-induced oxidative stress in neurons and PC12 cells.

Neurons are susceptible to acute oxidative stress^1^. Chronically elevated levels of reactive oxygen species (ROS) such as H_2_O_2_ have been implicated in neuronal cell death in many neurodegenerative disorders such as Alzheimer’s disease, Parkinson’s disease, Huntington’s disease, and amyotrophic lateral sclerosis^2^,^3^,^4^,^5^,^6^,^7^. ROS also contribute to acute damage resulting from cerebral ischemia^8^,^9^ and to genomic instability^10^,^11^. The accumulation of H_2_O_2_ induces apoptotic death in cultured neurons^12^ by damaging proteins and lipids and, especially, through accumulation of lesions in genomic and mitochondrial DNA^13^,^14^.

Protein kinase B (PKB)/Akt is one of the central regulators of neuronal survival^15^,^16^. Activation of Akt upon exposure to high glutamate^17^ or MPTP^18^ rescues primary neurons. H_2_O_2_-induced oxidative stress mediates phosphorylation of Akt to promote survival in neurons^19^,^20^. Moreover, activation of Akt signaling is neuroprotective against hypoxic and excitotoxic neuronal death *in vitro* and ischemic neuronal death *in vivo*^16^,^21^,^22^,^23^. In addition, cerebral ischemia induces neuronal death in part by inhibiting Akt activity^24^,^25^. Although accumulation of DNA strand breaks as a contributing factor to neurodegeneration during oxidative stress^26^ and the critical role of Akt in DNA damage-induced cell death signaling are recognized^27^,^28^, the mechanism by which Akt promotes neuronal survival during H_2_O_2_-induced cell death remains elusive.

An early response to the induction of DNA damage is post-translational modification of histones. Alterations in the capacity for DNA condensation or changes in the charge or modifications of histone proteins can modulate the binding interface for chromatin-associated proteins^29^. Since neurons do not replicate their DNA one of the first changes observed during apoptotic death is DNA condensation; therefore, specific histone modifications might be correlated with neuronal apoptosis. In this study, we demonstrated a new defense mechanism in neuronal cells that is regulated by Akt signaling during H_2_O_2_-induced oxidative stress. Under moderate oxidative stress Akt was activated and directly interacted with histone 2A, phosphorylating the N-terminal tail of H2A. This interaction activated pro-survival signaling and postponed apoptosis. However, upon continued oxidative stress the neurons failed to sustain Akt signaling and underwent apoptotic cell death. We found that Akt recruited Sirt2, a member of a family of conserved NAD^+^-dependent protein deacetylases that bound to Akt-mediated phosphorylated H2A and decreased H2A acetylation, suggesting that H2A is a new deacetylation target of Sirt2 in neuronal cells under H_2_O_2_-induced oxidative stress. Our findings indicate that Akt regulates neuronal apoptosis by fine-tuning H2A protein modification under conditions of H_2_O_2_-induced oxidative stress.

## Results

### Akt interacts with H2A

**Figure 1:**
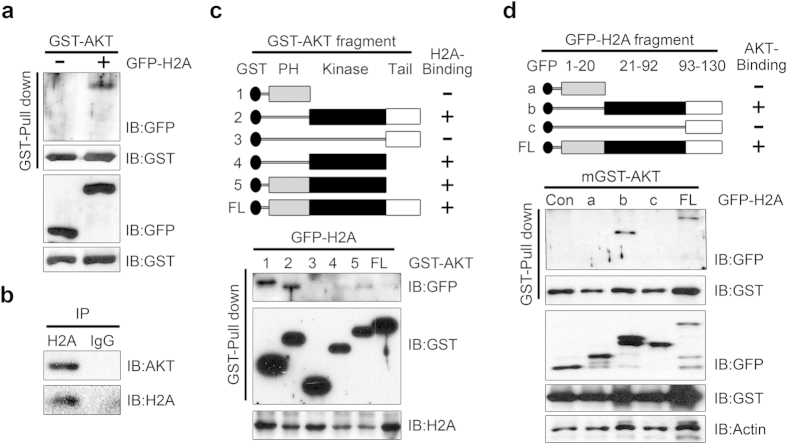
Akt interacts with H2A. (**a**) HEK 293T cells were co-transfected with GST-Akt and GFP-H2A. Cell extracts were immunoprecipitated with GST beads and immunoblotted with antibodies as indicated. (**b**) HEK 293T cells were harvested and lysed. Proteins were immunoprecipitated with anti-H2A antibody or normal IgG. (**c**) Schematic representation of GST-Akt full-length (FL) and fragment constructs used to identify the H2A interaction region in Akt (upper). 293T cells were transfected with GST-Akt full-length (FL) or fragments together with GFP-tagged H2A. Proteins were pulled down with GST resin and visualized by immunoblotting. (**d**) Schematic representation of GFP-H2A full-length (FL) and fragment constructs used to identify the Akt interaction region in H2A (upper). HEK 293T cells were transfected with GFP-H2A full-length (FL) or fragments with GST-AKT. Cells were analyzed as described above.

In our search for a target of active nuclear Akt we carried out immunoprecipitation assays with nuclear extracts from PC12 cells that constitutively expressed active Akt or mock vector as a control. Immunoprecipitated proteins were eluted and resolved by SDS-PAGE. Prominent protein bands were subjected to proteomic analysis and histone 2A was identified as a putative binding partner of Akt. Using GST pull-down analysis, we verified a specific interaction between GST-Akt and GFP-H2A (Fig. 1a). In intact cells, endogenous Akt bound to endogenous H2A (Fig. 1b). To ascertain the specificity of the binding between Akt and H2A we performed GST pull-down analysis with histone protein family members (H2A, H2B, H3, and H4) and found that H2A was the strongest binding partner of Akt (Supplementary Fig. S1). *In vitro* binding assays with a series of Akt fragments expressed as GST fusions in HEK 293 cells demonstrated that the catalytic domain of Akt was required for interaction with H2A, raising the possibility that H2A is a kinase substrate of Akt (Fig. 1c). Reciprocal mapping analysis with GFP-H2A fragments showed that the internal region is responsible for the interaction with Akt (Fig. 1d).

### H2A is a physiological substrate of Akt

**Figure 2:**
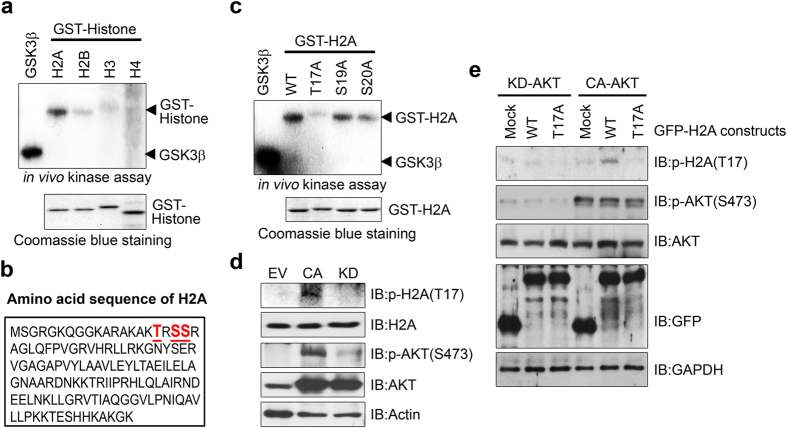
H2A is a physiological substrate of Akt. (**a**) GST-tagged histone proteins (H2A, H2B, H3, and H4) were bacterially expressed and purified using GST resin. A total of 500 ng of each protein was used for *in vitro* kinase assays with active Akt. The reaction products were separated by SDS-PAGE and exposed to film through autoradiography. GSK3 fusion protein (GSK-FP) was used as a positive control. (**b**) Schematic representation of the amino acid sequence of H2A. (**c**) GST-tagged histone H2A wild-type (WT) and mutant proteins (T17A, S19A, and S20A) were prepared and the *in vitro* kinase assay was performed as described above. (**d**) Cell extracts of PC12 cells expressing CA-Akt or KD-Akt were immunoblotted with anti-H2A-pT17 antibody. (**e**) PC12 cells expressing CA-Akt or KD-Akt were transfected with the indicated plasmids. Proteins were analyzed as described above.

Using *in vitro* kinase analysis with purified GST-histone proteins we found that, among histone family members, H2A was the most strongly phosphorylated by active Akt, consistent with our binding analysis showing that the strongest interaction between Akt and histone proteins occurred between H2A and Akt. This suggests that H2A is a prominent nuclear target of Akt (Fig. 2a and Supplementary Fig. S1).

Analysis of the amino acid sequence of H2A revealed the presence of several consensus sequence phosphorylation sites for Akt surrounding threonine 17, serine 19, or serine 20 in the amino terminus (Fig. 2b). We prepared a variety of recombinant GST-tagged H2A wild-type and mutant forms in which the putative phosphorylation residues were changed from threonine or serine to alanine and examined their abilities to be phosphorylated by Akt. *In vitro* kinase assays showed that wild-type H2A, H2A-S19A, and H2A-S20A mutant forms of H2A were substantially phosphorylated by Akt whereas H2A-T17A failed to be phosphorylated, indicating that T17 is selectively phosphorylated by Akt (Fig. 2c). Antibody that specifically recognizes phosphorylated H2A-T17 (H2A-pT17) gave a signal in PC12 cells expressing constitutively active (CA)-Akt whereas this signal was abolished in PC12 cells expressing kinase dead (KD)-Akt or control vector^30^ (Fig. 2d). Moreover, anti-H2A-pT17 antibody recognized phosphorylated H2A in cells that were cotransfected with H2A-WT and HA-CA-Akt (Fig. 2e). In contrast, this antibody failed to detect phosphorylated H2A in cells that were cotransfected with H2A-T17A and HA-CA-Akt. Furthermore, H2A-T17A phosphorylation was completely abolished by cotransfection with either WT-H2A or T17A-H2A and KD-Akt (Fig. 2e). These data indicate that H2A-pT17 is an *in vivo* substrate of Akt kinase in PC12 cells.

### Phosphorylation of H2A on T17 by Akt occurs during H_2_O_2_-induced cell death

**Figure 3:**
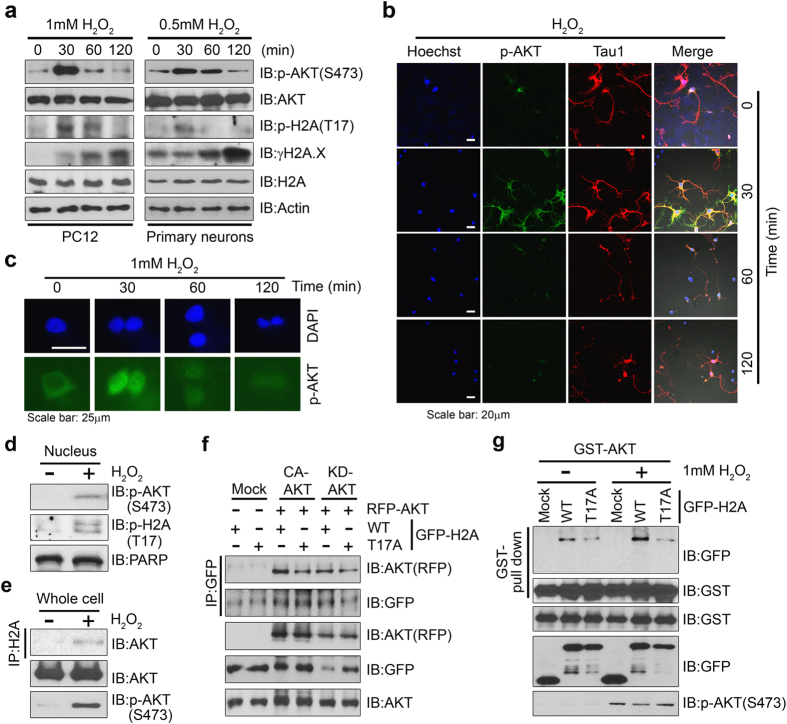
Phosphorylation of H2A on T17 by Akt occurs during H_2_O_2_-induced cell death. (**a**) PC12 cells (left) and primary cultured hippocampal neurons (right) were treated with 1 mM or 0.5 mM H_2_O_2_ respectively for 30, 60, and 120 min and immunoblotting was performed with the indicated antibodies. (**b**) Fixed hippocampal neuron cells were stained with Hoechst 33342 (blue), anti-pAkt antibody (green), and anti-Tau1 antibody (red). (**c**) PC12 cells were maintained without serum for 4 h, followed by H_2_O_2_ treatment. Fixed cells were stained with DAPI (blue) and anti-pAkt antibody (green). After H_2_O_2_ stimulation for 30 min, active Akt translocated from the cytoplasm into the nucleus. (**d**) Total nuclear proteins were isolated from PC12 cells treated with or without H_2_O_2_ and fractionated into cytoplasmic and nuclear proteins. Nuclear fractions were analyzed by immunoblotting with anti-pH2A antibody. PARP was used as a marker for the nuclear fraction. (**e**) PC12 cells were stimulated with 1 mM H_2_O_2_ for 30 min and cell lysates were used for immunoprecipitation with anti-H2A antibody. The association between endogenous Akt and H2A was enhanced by H_2_O_2_ treatment. (**f**) RFP-Akt constructs (active Akt T308DS473D or kinase-dead K179A) were co-transfected with GFP-tagged H2A-WT and mutant (H2A-T17A) into HEK 293T cells. Cell lysates were analyzed by immunoblotting with the indicated antibodies. (**g**) GST-Akt and GFP-H2A constructs (WT and mutant H2A-T17A) were co-transfected into PC12 cells. After 24 h, cells were stimulated with 1 mM H_2_O_2_ for 30 min and cell lysates were used for co-precipitation with glutathione beads. The association between exogenous Akt and H2A-WT was enhanced by H_2_O_2_ treatment.

Because Akt phosphorylation was most dramatically upregulated by H_2_O_2_ treatment among various genotoxic insults that we tested (Supplementary Fig. S2), and it has been reported that phosphorylation of histones is linked to hydrogen peroxide-induced apoptosis^31^, we wondered whether Akt activation during hydrogen peroxide-induced DNA damage is linked to phosphorylation of H2A-T17. We first examined the pattern of Akt activation upon H_2_O_2_ treatment of PC12 cells. With increasing time of incubation with 1 mM H_2_O_2_ Akt phosphorylation reached a maximal state at 30 min and then declined to a basal level after 2 h (Fig. 3a left, first panel). Concordant with Akt activation, levels of H2A-pT17 were robustly increased at 30 min and sustained for up to 2 h (Fig. 3a left, third panel). Moreover, when we extended the H_2_O_2_ exposure time to 4 h we similarly observed maximal activation of Akt at 30 min, which gradually decreased over 2 h and was completely abolished at 4 h (Supplementary Fig. S3 and Fig. 3a left), implying that prolonged exposure to H_2_O_2_ induced irreversible DNA damage. Indeed, we detected increased expression of phospho-γ-H2AX, a well-known marker of DNA damage during cell death, which was inversely correlated with the Akt phosphorylation state (Fig. 3a left, fourth panel). Moreover, we confirmed that Akt phosphorylates H2A during 0.5 mM H_2_O_2_-induced DNA damage in primary cultured hippocampal neurons (Fig. 3a right). Immunostaining analysis also demonstrated a higher signal intensity for phosphorylated Akt in primary hippocampal neurons at 30 min after H_2_O_2_ exposure, which had diminished by 2 h (Fig. 3b).

Consistent with our previous report that phosphorylated active Akt translocates into the nucleus in neuronal cells^32^, we found that phosphorylated Akt was intensely detected in the nucleus after 30 min of H_2_O_2_ treatment (Fig. 3c) where it presumably acts as a kinase for its nuclear targets such as H2A. The results of a subcellular fractionation assay demonstrated Akt nuclear translocation and subsequent phosphorylation of H2A-T17 after 30 min exposure to H_2_O_2_ (Fig. 3d). Thus, activation of Akt and its nuclear localization are essential for the phosphorylation of T17 on H2A during H_2_O_2_ insult before the cells undergo death.

In accordance with our finding that Akt phosphorylates H2A upon H_2_O_2_-induced oxidative stress, we found that activation of Akt enhanced its physical interaction with H2A (Fig. 3e) and it appeared that both Akt activation and Akt-mediated phosphorylation of H2A were required for their interaction (Fig. 3f). Constitutively active (CA)-Akt associated with H2A-WT more strongly than with kinase dead (KD)-Akt, indicating that Akt activation is necessary for the interaction between Akt and H2A (Fig. 3f, first panel lanes 3 and 5). The association between CA-Akt and H2A-T17A or between KD-Akt and H2A-T17A showed a basal level of interaction, implying that Akt-mediated H2A phosphorylation is required for its interaction with Akt (Fig. 3f, first panel, lanes 4 and 6). The results of GST pull-down analysis provided further evidence that Akt-mediated phosphorylation of H2A affects the interaction between Akt and H2A. In the absence of H_2_O_2_ treatment, H2A-T17A, which cannot be phosphorylated by Akt, barely bound to Akt whereas H2A-WT strongly interacted with Akt. Moreover, H_2_O_2_ treatment failed to induce binding of H2A-T17A to Akt but notably enhanced the interaction between H2A-WT and Akt (Fig. 3g), indicating that phosphorylation of H2A on T17 is required for its association with active Akt.

### Akt-mediated phosphorylation of H2A on T17 delays apoptotic death in neurons

**Figure 4:**
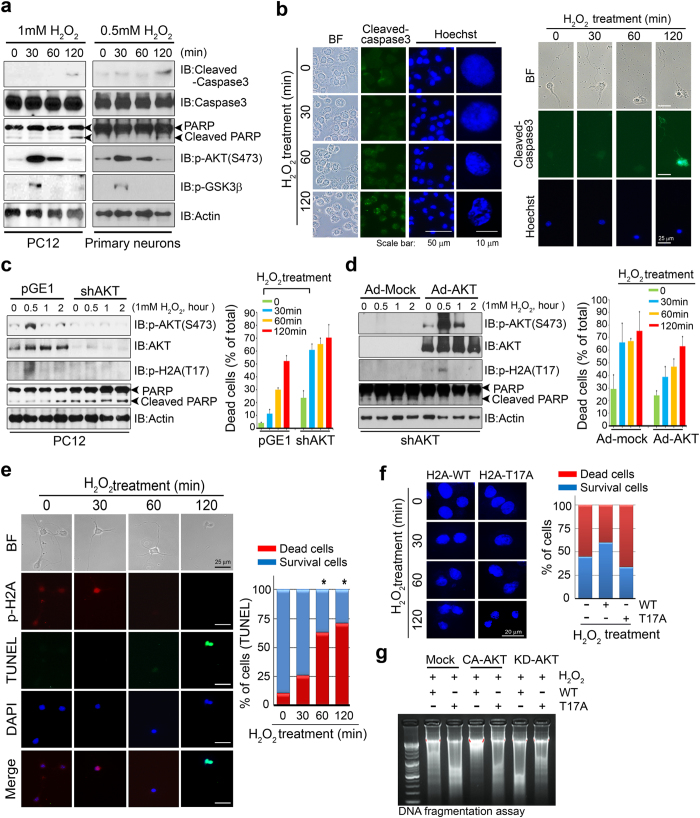
Akt-mediated phosphorylation of H2A on T17 delays apoptotic death in neurons. (**a**) PC12 cells and primary cultured hippocampal neurons were treated with H_2_O_2_ (1 mM and 0.5 mM respectively) and cell extracts were immunoblotted with the indicated antibodies. (**b**) PC12 cells and primary hippocampal neurons were treated with H_2_O_2_ (1 mM and 0.5 mM respectively) for 30, 60, and 120 min. Cleaved caspase-3 was detected at 120 min. Fixed cells were stained with Hoechst 33342 (blue) and anti-cleaved caspase-3 antibody (green). Figures on the right indicate apoptotic cells with condensed chromatin morphologies. (**c**) PC12 cells were transfected with pGE-sh-Akt and pGE-mock (control) for 24 h and then exposed to H_2_O_2_ (1 mM) for the indicated time, followed by immunoblotting and quantitative analysis of cell death was determined by Hoechst staining. (**d**) PC12 cells, transfected with pGE-sh-Akt and pGE-mock (control), were infected with Adenovirus-GFP-Akt and then exposed to H_2_O_2_ (1 mM) for the indicated time, followed by immunoblotting and quantitative analysis of cell death was determined by Hoechst staining. (**e**) Primary hippocampal neurons were stained with anti-H2A-p17 antibody (red) after H_2_O_2_ exposure for the indicated time. Apoptotic cells measured by TUNEL assay are presented in the bar graph (bottom). *p < 0.05. (**f**) Primary hippocampal neurons were transfected with H2A-WT or H2A-T17A and exposed to H_2_O_2_. Fixed cells were stained with Hoechst 33342 (blue). Apoptotic cells with condensed chromatin morphologies measured by cell counting assay are presented in the graph. (**g**) PC12 cells were co-transfected with CA-Akt (or KD-Akt) and H2A-WT (or mutant construct), followed by H_2_O_2_ treatment for 120 min. Genomic DNA was separated on a 2% agarose gel. The percentage of cell death was determined at the indicated time point after H_2_O_2_ treatment. At least three separate experiments were performed and the results are the means of three independent experiments.

Previously, we reported that nuclear Akt is a central regulator of neuronal survival by inhibiting apoptosis^30^,^33^ and promotes cell survival by phosphorylation of ribosomal protein S3 in response to DNA insults^34^. As our data showed that H_2_O_2_ exposure of primary neurons and PC12 cells induced substantial activation of Akt at 30 min that subsequently decreased within 2 h (Fig. 3a left and right), we tested whether Akt activation is involved in protecting against apoptotic cell death under conditions of DNA damage. As shown in both PC12 cells (left) and hippocampal neurons (right), apoptotic cell death, as revealed by the hallmarks of cleaved caspase 3 and poly (ADP-ribose) polymerase (PARP) (Fig. 4a, first and third panel), immunoreactivity of cleaved caspase-3 (Fig. 4b), and chromatin condensation, was observed concurrently with the decline in Akt activation after 2 h of H_2_O_2_ treatment (Fig. 4a, fourth panel) in both PC12 cells and primary cultured hippocampal neurons. However, at 30 min of H_2_O_2_ treatment, when Akt was activated, apoptosis was inhibited and the cells possessed intact nuclei (Fig. 4b). The subsequent decrease in Akt activation was confirmed by Akt phosphorylation status and phosphorylation levels of GSK3-β, a recognized substrate of Akt (Fig. 4a, fourth and fifth panel). To confirm a direct link between Akt activation and attenuation of apoptosis during H_2_O_2_-induced oxidative damage, we depleted Akt from PC12 cells using sh-Akt. Silencing of Akt abolished H2A phosphorylation. Importantly, silencing the expression of Akt elicited caspase-3 activation as assessed by PARP cleavage and immunostaining of cleaved caspase-3 in the absence of any insult and obviously provoked apoptotic death after H_2_O_2_ treatment, thus removing resistance to H_2_O_2_-induced oxidative stress. At 30 min of H_2_O_2_ treatment in the absence of Akt, more than 60% of cells underwent cell death while control cells were still resistant for death in the presence of Akt and subsequent phosphorylation of H2A by Akt (Fig. 4c and Supplementary Fig. S4a). Reintroduction of adenovirus-GFP-Akt into Akt-depleted cells restored the resistance to H_2_O_2_-induced apoptosis and the associated H2A phosphorylation (Fig. 4d and Supplementary Fig. S4b and S4c). These data led us to consider Akt activation after H_2_O_2_ treatment as an immediate defense system to prevent apoptotic death whereby cellular survival is favored before the accumulation of irreversible DSBs.

As activated Akt phosphorylated H2A and tightly associated with H2A in the nucleus during H_2_O_2_ exposure, we investigated whether Akt provoked survival signaling through the phosphorylation of H2A. Primary cultured hippocampal neurons were double-stained with TUNEL and anti-phosphoT17-H2A antibody and examined by immunofluorescence microscopy (Fig. 4e). In accordance with the loss of viability, after H_2_O_2_ treatment for 30 minutes approximately 25% of WT cells showed TUNEL staining, a hallmark of apoptosis, whereas after 2 h more than 75% of cells showed TUNEL positivity (Fig. 4e bottom and Supplementary Fig. S4d). Strikingly, TUNEL-positive cells were not co-stained with anti-phospho-H2A-T17 antibody, demonstrating that apoptotic DNA fragmentation inversely correlates with H2A phosphorylation at T17 (Fig. 4e).

Chromatin condensation and DNA fragmentation are the most prominent nuclear features during apoptotic cell death. Primary hippocampus neurons were transfected with H2A-WT or H2A-T17A. The nuclei of transfected cells were stained with Hoechst 33342 following a time course of H_2_O_2_ treatment. Neurons transfected with H2A-WT showed intact nuclei by Hoechst staining at 30 min after H_2_O_2_ treatment, followed by an increase in shrunken and dense nuclear bodies as exposure time increased. In contrast, neurons expressing mutant H2A-T17A displayed remarkable chromatin condensation by Hoechst staining, revealing less than 30% cell viability (Fig. 4f). In addition, genomic DNA fragmentation analysis demonstrated that H2A-WT expression inhibited DNA fragmentation under H_2_O_2_ treatment in the presence of CA-Akt whereas H2A-T17A failed to prevent fragmentation. However, in the presence of KD-Akt neither H2A-WT nor H2A-T17A inhibited DNA fragmentation (Fig. 4g). Thus, our data suggest that Akt-mediated phosphorylation of H2A provides an early defense system from DNA damage for neurons before the generation of severe DSBs.

**Figure 5:**
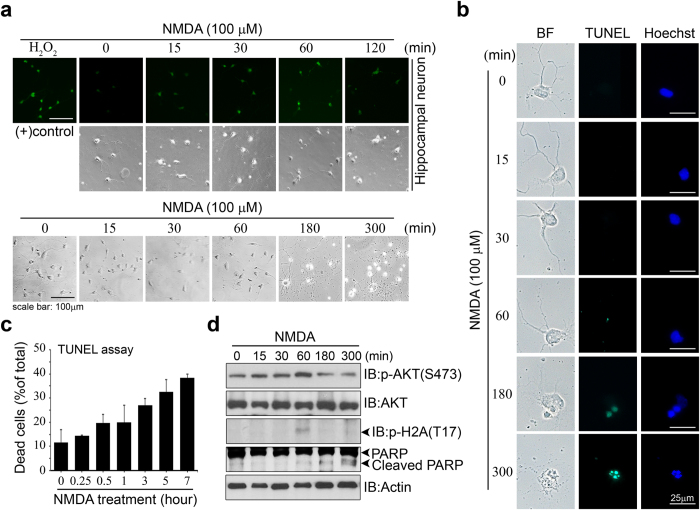
NMDA excitotic cell death reflects H_2_O_2_ mediated Akt/H2A signaling. (**a**) Hippocampal neuron cells were treated with 100 μM NMDA for 0 to 2 hours. Afterwards, cells were incubated with CM-H_2_DCFDA for measuring H_2_O_2_ production. Cell image was obtained using a fluorescent microscope (upper). Cells were treated with H_2_O_2_ for 10min as positive control. Primary neuron cultures were treated with NMDA (100 μM) for 5 hours and representative digital image of cells are seen in the bottom of Fig. 5a. Scale bar, 100 μm.(**b**) Primary hippocampal neurons were stained with TUNEL (green) after NMDA treatment for the indicated time. Scale bar, 25 μm. (**c**) Apoptotic cells measured by TUNEL assay are presented in the bar graph. (**d**) Primary cultured hippocampal neurons were treated NMDA (100 μM) and cell extracts were immunoblotted with indicated antibodies.

We next sought to confirm our results using an excitotoxic model of *N*-methyl-D-aspartate (NMDA) receptor overstimulation that simulates endogenous H_2_O_2_-induced pathophysiology^35^,^36^. Excitotoxicity triggers cell death, which shares many features of apoptosis, such as cell body shrinkage, nuclear condensation, and DNA fragmentation^37^. Primary cultured neurons were treated with NMDA (100 μM) for 0, 15, 30, 60, 120, 180, and 300 min (or further time) and the generation of endogenous H_2_O_2_ was determined by CM-H_2_DCFDA staining (Fig. 5a upper). We examined Akt activation, which was monitored phosphor Akt levels in hippocampal neurons subjected to NMDA induced neurotoxicity and subsequent phosphorylation of H2A. Treatment of primary cultured neurons elicited cell death as evidenced by morphological alteration, nuclear morphology and Tunel assay (Fig. 5a bottom, b,c). In this hippocampal cultures, phosphor Akt levels initially increased at 60 min after NMDA treatment followed by a decline evident at 180 min (Fig. 5d), with negligible levels after 15 h (data not shown). The levels of H2A T17 phosphorylation in hippocampal neurons after NMDA treatment was increased at 60 min and declined with a time course similar to that of the decline in levels of phosphor Akt (Fig. 5d). Although time frame of Akt activation and subsequent activation of Akt/H2A signaling was a bit shifted, our data indicate that NMDA excitotoxic cell death reflects endogenous H_2_O_2_ production and consequent activation of Akt/H2A signaling delaying neuronal death at early time point of oxidative stress induced cell death.

### Akt recruits Sirt2 for H2A deacetylation

**Figure 6:**
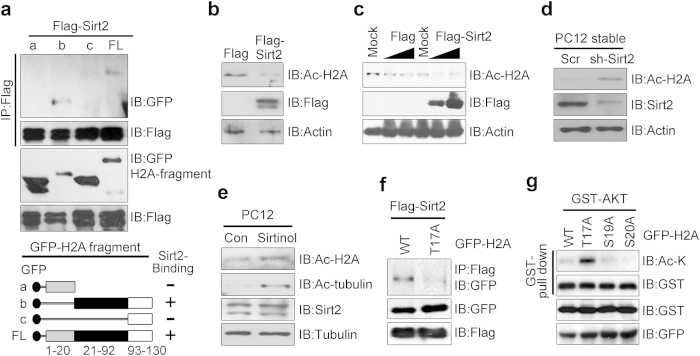
Akt recruits Sirt2 for H2A deacetylation. (**a**) 293T cells were co-transfected with FLAG-SIRT2 and GFP-H2A fragments. Cell extracts were immunoprecipitated with anti-FLAG antibody and immunoblotted with the antibodies indicated. Schematic representation of H2A fragments and constructs used to identify the SIRT2 interaction region in H2A (lower). (**b**) 293T cells were transfected with the indicated plasmids for 24 h, and equal amounts of cell lysate were subjected to immunoblotting using the indicated antibodies. (**c**) PC12 cells were transfected with Mock and FLAG-SIRT2 constructs in a dose-dependent manner. The cell extracts were immunoblotted with anti-acetyl H2A (K5) antibodies as indicated. (**d**) PC12 cells with stable knockdown of SIRT2 were harvested and lysed. Proteins were immunoblotted with anti-acetyl H2A (K5). (**e**) PC12 cells were treated with Sirtinol (50 μm for 24 h) and immunoblotted with the indicated antibodies. (**f**) PC12 cells were co-transfected with FLAG-SIRT2 and GFP-H2-WT (or H2A-T17A) constructs. The cell extracts were immunoprecipitated with anti-FLAG antibody and immunoblotted with the antibodies indicated. (**g**) PC12 cells were co-transfected with GST-Akt and GFP-tagged H2A constructs (WT, T17A, S19A, and S20A). Proteins were pulled down with GST resin and visualized by immunoblotting.

Because terminally differentiated cells like neurons do not replicate their DNA, removal of DNA damage from the nonessential bulk of their genome is dispensable and neurons can afford to repair only the portion of the genome needed for their function, such as the transcribed DNA^38^,^39^. Histone acetylation is generally associated with transcriptional activation whereas deacetylation is linked with transcriptionally silent heterochromatin^40^,^41^. A recent study showed that the NAD^+^-dependent deacetylase sirtuin 2 (Sirt2) binds to Akt through the pleckstrin homology (PH) and kinase domains of Akt and maintains Akt activation^42^. Given that H2A also interacts with the PH and kinase domains of Akt (Fig. 1d) we wondered whether Sirt2 is involved in the modulation of Akt-mediated phosphorylation of H2A in neuronal apoptosis. Our mapping analysis demonstrated that Sirt2 interacts with an internal domain of H2A, implying that these three molecules might form a triple complex in cells (Fig. 6a).

We next evaluated whether Sirt2 is responsible for H2A deacetylation. Overexpression of Flag-Sirt2 markedly diminished endogenous H2A acetylation, and Sirt2-mediated deacetylation of H2A occurred in a dose-dependent manner in PC12 cells (Fig. 6b,c). To further determine whether Sirt2 physiologically acts as deacetylase for H2A we generated a knockdown cell line of Sirt2 using lentiviral sh-Sirt2 (Supplementary Fig. S5). Silencing of Sirt2 in PC12 cells elicited increased levels of acetylation on H2A (Fig. 6d). Using Sirtinol, a chemical inhibitor of Sirt2, we further demonstrated that inhibition of the deacetylase activity of Sirt2 resulted in enhanced acetylation of H2A, indicating that Sirt2 is a deacetylase of H2A (Fig. 6e, first panel). The activity of Sirtinol was confirmed by determining the acetylation levels of tubulin (Fig. 6e, second panel).

To examine whether phosphorylation of T17 in H2A by Akt affects the association between H2A and Sirt2, we co-transfected cells with Flag-Sirt2 and GFP-H2A-WT or H2A-T17A. Our immunoprecipitation assay showed that H2A-WT interacted with Sirt2 whereas H2A-T17A minimally bound to Sirt2, suggesting that H2A-T17 phosphorylation is required for the association with Sirt2 (Fig. 6f). Our *in vitro* GST pull-down assay with purified GST-H2A-WT and point mutants including T17A demonstrated that H2A-T17A was highly acetylated whereas H2A-WT and other mutants that were able to be phosphorylated showed rare acetylation (Fig. 6g). These data suggest that H2A-WT might be a substrate of Sirt2 when it binds to Akt and is phosphorylated since H2A-T17A, which barely binds Akt and cannot be phosphorylated by Akt, is not an available substrate for Sirt2. It is conceivable that under mild DNA damage conditions Akt rapidly phosphorylates H2A on T17 and prohibits chromatin condensation whereas under lethal damage Akt provides phospho-H2A for deacetylation by Sirt2 to ensure apoptotic cell death.

## Discussion

The discovery of novel histone modifications initiated a major leap forward in our understanding of how cells regulate DNA metabolic events such as transcription, replication, and DNA damage repair and how these mechanisms are interlinked with cell death and act in concert in response to DNA damage. However, because of the unique post-mitotic nature of neurons it was unclear how neurons with DNA damage die. In this report, we uncover a previously unrecognized defense and death function of H2A modification during H_2_O_2_-induced DNA damage. We identified a specific interaction between Akt and H2A and demonstrated T17 phosphorylation of H2A by Akt under oxidative stress. Phosphorylation of T17 in H2A by Akt plays a defense role against neuronal apoptosis under moderate DNA damage but rapidly serves as a death signal by acting as a substrate of Sirt2 deacetylase as the H_2_O_2_-induced DNA damage becomes irreversible DSBs.

Previously, we have shown that Akt phosphorylates ribosomal protein S3 (RPS3), which acts as a proapoptotic protein, and enhances its endonuclease activity in the nucleus in response to DNA damage thereby promoting neuronal survival^34^. As we showed that Akt is present in the nucleus and activated to some extent in response to H_2_O_2_ treatment, and that H2A phosphorylation is correlated with Akt phosphorylation in a time-dependent manner, it is conceivable that Akt promotes cell survival by phosphorylating histone protein in the nucleus to give the cell some time to avoid death. Indeed, our study of the kinetics of apoptotic events and the onset of H2A-T17 phosphorylation showed that Akt activation and H2A-T17 phosphorylation preceded γ-H2A.X phosphorylation, which represents irreversible DNA double-strand break damage. However, as the amount of phosphorylated γ-H2A.X increased, Akt activation decreased and H2A phosphorylation was accordingly diminished. γ-H2A.X phosphorylation started to appear at 30 min of H_2_O_2_ treatment and intensely increased after 2 h, suggesting that H2A phosphorylation by Akt delays the generation of DNA double-strand breaks and the loss of phosphorylation of H2A is a critical signal for acquiring phosphorylation of γ-H2A.X (Fig. 4a). In addition, our time course analysis indicated that the onset of H2A phosphorylation was discordant with the appearance of DNA fragmentation evaluated by TUNEL staining (Fig. 4e). Moreover, ectopic expression of the H2A-T17A mutant, even in the presence of higher levels of endogenous wild-type H2A, robustly enhanced DNA fragmentation and TUNEL staining intensity (Fig. 4f,g), correlating with reduced interaction with active Akt. Furthermore we demonstrated that overstimulation of NMDA induced neurotoxicity elicits endogenous H_2_O_2_ production and consequent activation of Akt/H2A signaling resembling H_2_O_2_ insult, delaying neuronal death at early time point of oxidative stress induced cell death (Fig. 5a–d). Therefore this H2A modification may function to facilitate neuronal survival precedent to irreversible DNA damage under conditions of oxidative stress. Our data suggest that H2A phosphorylation by Akt may play a role in resisting apoptotic DNA fragmentation early in DNA damage signaling, thus detaining cells for survival.

Regulating the ability for DNA condensation or altering the charge or modification of histone proteins can modulate the binding interface for chromatin-associated proteins^29^. Post-translational modification of histones is an early response to the induction of DNA damage and can provide unique binding sites for interacting factors. Interestingly, we found that under conditions of oxidative stress the PH and kinase domain of Akt is available for binding of not only H2A but also the deacetylase Sirt2 (Figs 1c and 6a), suggesting that these three molecules could form a triple complex in cells and in mouse brain lysates. T17-phosphorylated H2A interacts more strongly with both Akt and Sirt2 (Figs 3d,e and 6f), whereas the H2A-T17A mutant rarely interacts with either Akt or Sirt2 (Figs 3g and 6f). In accordance with this observation H2A is deacetylated by Sirt2 but H2A-T17A is not, suggesting that H2A phosphorylation is required as a marker for Sirt2-mediated deacetylation, which in turn might be responsible for the DNA fragmentation and chromatin condensation leading to apoptosis when DNA damage is severe. Our data suggest that modification of H2A by Akt may be a general stress response pathway to promote neuronal survival in early damage conditions that switches to deacetylation for later induction of apoptotic death. Clearly, a better understanding of how such modifications of histone proteins change signaling from prosurvival to cell death pathways is required.

## Materials and Methods

### Cell culture

PC12 cells were maintained in Dulbecco’s modified Eagle’s medium (DMEM) with 10% fetal bovine serum, 5% horse serum, and 100 units of penicillin/streptomycin at 37 °C under a 5% CO_2_ atmosphere. HEK 293T cells were maintained in DMEM supplemented with 10% fetal bovine serum at 37 °C under a 5% CO_2_ atmosphere. PC12 cells and HEK293T cells were used for no more than 10 and 20 passages, respectively. Cells were transfected by electroporation. For primary hippocampal neuron culture, the hippocampus was dissected from a newborn rat. After trypsinization, the cells were cultured in neurobasal-A/B27 media at 37 °C under a 5% CO_2_ atmosphere in a humidified incubator^43^.

### Antibodies, plasmids, and chemicals

Anti-histone H2A, anti-pan Akt, anti-acetylated lysine, and anti-phospho-GSK3ß antibodies were obtained from Cell Signaling Technology (Danvers, MA, USA). Anti-GFP, anti-GST, anti-HA, anti-actin, and anti-tubulin antibodies were acquired from Santa Cruz Biotechnology (Santa Cruz, CA, USA). All other chemicals were obtained from Sigma (St. Louis, MO, USA). Polyclonal anti-phospho-H2A Thr17-specific antibodies were generated by injecting synthesized Thr17-phosphorylated peptide (SGRGKQGCKARAKAKtRSS) into rabbits. Antibodies were affinity purified from serum using unphosphorylated peptide cross-linked to Affi-Gel 15 according to the manufacturer’s recommendations (AbClon, Seoul, Korea). H2A was cloned into pEGFP-C2 and pGEX4T1 vectors. PCR-based mutagenesis was performed using the QuikChange site-directed mutagenesis kit (Stratagene, La Jolla, CA, USA) according to the manufacturer’s instructions using the following primers: T17A forward, 5′-CGCGCCAAGGCTAAGgCTCGGTCTTCTCGTGCAGGT TTGCAG-3′, and reverse, 5′-CTGCAAACCTGCACGAGAAGACCGAGcCTTAGCCTTGGCGCG-3′). Oligomers for shAkt1 (shAkt1 forward, 5′-CCATGAACGAGTTTGAGTACC-3′, and reverse, 5′-GGTACTCAAACTCGTTCATGG-3′) were cloned into a pGE1 vector. The Akt was inserted into adenoviral vector using Ad-Easy-System (Stratagene, San Diego, CA, USA). Sh-SIRT2 was cloned into FLAG vector (Addgene, Cambridge, MA, USA). Sirtinol was obtained from Selleckchem (Houston, TX, USA).

### Co-immunoprecipitation assay and *in vitro* binding assay

For co-immunoprecipitation, cells were rinsed with phosphate-buffered saline (PBS) and lysed in buffer (50 mM Tris-Cl, pH 7.4, 150 mM NaCl, 1 mM EDTA, 0.5% Triton X-100, 1.5 mM Na_3_VO_4_, 50 mM sodium fluoride, 10 mM sodium pyrophosphate, 10 mM beta-glycerolphosphate, 1 mM phenylmethlysulfonyl fluoride [PMSF], and protease cocktail [Calbiochem, San Diego, CA, USA]). Cell lysates (0.5 to 1 mg of protein) were mixed with primary antibody and protein G/A beads and incubated for 3 h at 4 °C with gentle agitation. The beads were then washed in lysis buffer, mixed with 2× SDS sample buffer, boiled, and analyzed by immunoblotting. For the *in vitro* binding assay, proteins were expressed in bacteria, purified with GST resin, and dialyzed to PBS. For binding, 500 ng of intact protein was reacted at 4 °C for 1 h with gentle agitation and immunoprecipitated with the indicated antibodies.

### GST pull-down assay

Cells were rinsed with PBS and lysed in buffer as described above. Cell lysates (0.5 to 1 mg of protein) were mixed with glutathione-Sepharose beads and incubated for 3 h at 4 °C with gentle agitation. The beads were washed in lysis buffer, mixed with 2× SDS sample buffer, boiled, and analyzed by immunoblotting^44^.

### *In vitro* kinase assay

Purified protein (0.5 μg) was incubated with recombinant active Akt (Upstate) and 10 μCi of gamma ^32^P-ATP (Perkin-Elmer, Branchburg, NJ, USA) in 50 μl of kinase buffer (25 mM Tris-HCl [pH 7.5], 5 mM beta-glycerophosphate, 2 mM dithiothreitol [DTT], 0.1 mM Na_3_VO_4_, 10 mM MgCl_2_, and 0.2 mM ATP). Reactions were incubated at 30 °C for 30 min and terminated by addition of Laemmli SDS sample buffer and boiling at 95 °C for 5 min. Proteins were separated on NuPAGE 4–12% gradient gels (Invitrogen) and phosphorylation of proteins was visualized by autoradiography.

### Immunostaining

Cells grown on coverslips in 24-well plates were fixed in PBS containing 4% paraformaldehyde for 15 min, permeabilized in PBS containing 0.25% Triton X-100 for 10 min, and blocked in 2% BSA for 30 min. Cells were immunostained using antibodies against p-AKT (ser473) with Alexa Fluor 488 goat anti-rabbit secondary antibody. Nuclei were counterstained with DAPI. Cells were visualized under a fluorescence microscope or a Zeiss LSM confocal fluorescence microscope (Jena, Germany)^45^.

### DNA fragmentation assay

Oligonucleosomal fragmentation of genomic DNA was determined as described previously^46^. Briefly, 3 × 10^6^ cells in 10 ml of medium were incubated with 1 mM H_2_O_2_ or H_2_O for 2 h. After incubation, the cells were lysed on ice for 60 min in 500 μl of lysis buffer (0.02% SDS/1% Nonidet P40/0.2 mg/ml proteinase K in PBS). Genomic DNA was extracted by the phenol/chloroform method and the pellet was dissolved in 50 μl of TE buffer (containing 10 mg/ml RNase) for 2 h at 37 °C. A total of 10 μg of DNA was electrophoresed on a 2% agarose gel and visualized under UV light.

### Subcellular fractionation (nucleus and cytoplasm)

Cells (1 × 10^6^ to 5 × 10^6 ^cells) were washed once with ice-cold PBS and once with lysis buffer (10 mM Hepes, pH 7.9, 10 mM KCl, 1.5 mM MgCl_2_, 0.5 mM PMSF, protease inhibitor mixture). Cells were lysed in 200 μl of lysis buffer containing 0.1% Nonidet P-40 for 10 min on ice. The lysates were centrifuged for 2 min at 5,000 × g at 4 °C, and the nuclear pellet was washed with lysis buffer without Nonidet P-40. Nuclei were resuspended in 40 μl of nuclear protein extraction buffer (10 mM Hepes, pH 7.9, 420 mM NaCl, 1.5 mM MgCl_2_, 0.2 mM EDTA) for 10 min at 4 °C. The nuclear fraction was cleared by centrifugation at 14,000 rpm at 4 °C for 10 min. Protein concentration was measured by Bradford assay^47^.

### Measurement of Intracellular H_2_O_2_ Levels

Endogenous H_2_O_2_ generation in primary neuronal cells was accessed by staining cells with 5-(and-6) chlolomethyl-2′,7′-dichlorfluorescein-diacetate (CM-H_2_DCFDA; Molecular Probes, Eugene, OR). Briefly, primary cultured neuron cells grown on coverslips in 24 well plate and cells were treated with NMDA in a time dependent manner. Cells were washed with fresh 1X PBS and incubated with 2 μM CM-H_2_DCFDA in serum-free media at 37 °C for 30 minutes according to manufacturer’s instruction. H_2_O_2_ treatment was used as a positive control. Cells were treated with 500 μM H_2_O_2_ before staining with CM-H_2_DCFDA. Subsequently, cells were fixed with 2% paraformaldehyde, and images were captured with fluorescence microscope. Three fields from each of the three separate wells were measured for each group.

### Cell death and TUNEL assays

Cells were cultured on coverslips (10^4^ cells/coverslip) and either treated with hydrogen peroxide (1 mM) or left untreated (controls). Cells were then stained with 4′,6-diamidino-2-phenylindole (DAPI) or subjected to TUNEL assays according to the manufacturer’s protocol (Calbiochem, San Diego, CA, USA). Cells with condensed nuclei or positivity for TUNEL staining were counted as positive for cell death. More than 200 random cells were counted for each individual sample. The experiments were repeated in triplicate and data are presented as the mean ± standard deviation (s.d.) (*p < 0.05, Student’s t-test).

### Lentivirus production and generation of stable lentivirus-expressing cell line

Lentivirus was generated by transfection with the piLenti Sirt2 siRNA-GFP plasmid (ABM #i029063d). Plasmid transfections were performed using lipofectamine 2000 transfection reagent (Invitrogen) according to the manufacturer’s instructions. Lentiviral media were harvested 72 h after transfection and filtered through a 0.45-μm syringe filter. PC12 cells were infected with Sirt2 siRNA lentiviral particle solution containing 8 μg/mL polybrene. After incubation in medium containing puromycin (1–2 μg/ml) for 2 or 3 weeks, puromycin-resistant cells were isolated and expanded.

## Additional Information

**How to cite this article**: Park, J. H. *et al*. Akt attenuates apoptotic death through phosphorylation of H2A under hydrogen peroxide-induced oxidative stress in PC12 cells and hippocampal neurons. *Sci. Rep*. **6**, 21857; doi: 10.1038/srep21857 (2016).

## Supplementary Material

Supplementary Information
