# Supplementary material for: Akt attenuates apoptotic death through phosphorylation of H2A under hydrogen peroxide-induced oxidative stress in PC12 cells and hippocampal neurons
Source: Sci Rep. 2016 Feb 22;6:21857. doi: 10.1038/srep21857 (PMC4761890; doi:10.1038/srep21857)

Supplementary Information

Akt attenuates apoptotic death through phosphorylation of H2A under hydrogen peroxide-induced oxidative stress in PC12 cells and hippocampal neurons

Ji Hye Park[1,3], Chung Kwon Kim[1,3], Sang Bae Lee[1], Kyung-Hoon Lee[2,3], Sung-Woo Cho[4] and Jee-Yin Ahn[1,3]*

[1]Department of Molecular Cell Biology, [2]Department of Anatomy and Cell Biology, [3] Center for Molecular Medicine, Samsung Biomedical Research Institute, Sungkyunkwan University School of Medicine, Suwon 440-746, Korea

[4]Department of Biochemistry and Molecular Biology, University of Ulsan, College of Medicine, Seoul 138-736, Korea.

*Correspondence should be addressed to: Jee-Yin Ahn, Department of Molecular Cell Biology, Sungkyunkwan University School of Medicine, 2066, Seobu-ro, Jangan-gu, Suwon 440-746, Korea. Phone: 82-31-299-6134 ; Fax: 82-31-299-6139 ; E-mail: jeeahn@skku.edu

Supplementary Figures

Figure S1 293T cells were co-transfected with GST-Akt and GFP-histone constructs. Cell extracts were incubated with GST beads and pull-down was conducted using GST-tagged fusion protein. Although various histone protein family members bound to Akt, a stronger interaction was observed for H2A compared with other histone proteins such as H2B, H3, and H4.

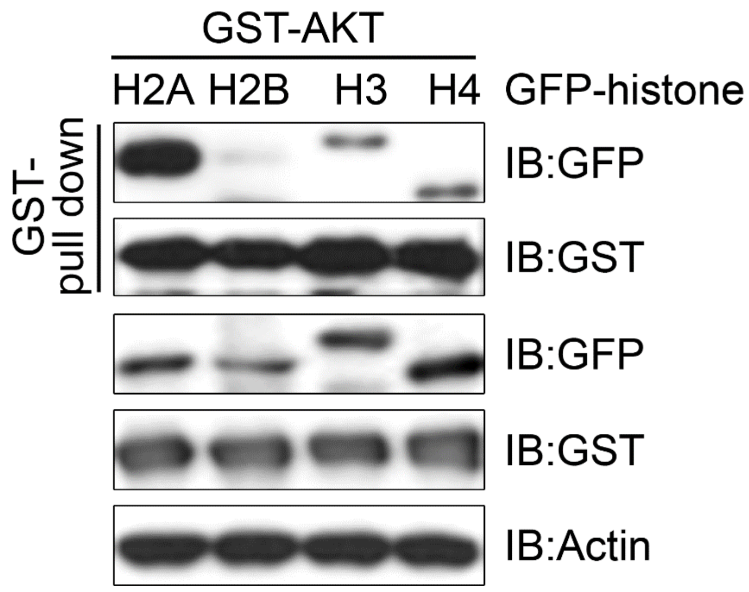

Figure S2 PC12 cells were stimulated by various growth factors and types of DNA damage. PC12 cells were starved for 6 h followed by treatment with NGF (100 ng/ml for 30 min), γ-irradiation (10 Gy after 30 min), UV irradiation using Stratagene UV Stratalinker 2400 Crosslinker (15 J/m^2 for 30 min), hydrogen peroxide (1 mM for 30 min), etoposide (50 μm for 6 h), or staurosporine (250 nM for 30 min). NGF and H_2O_2 increased the level of pH2A or pAkt in PC12 cells.

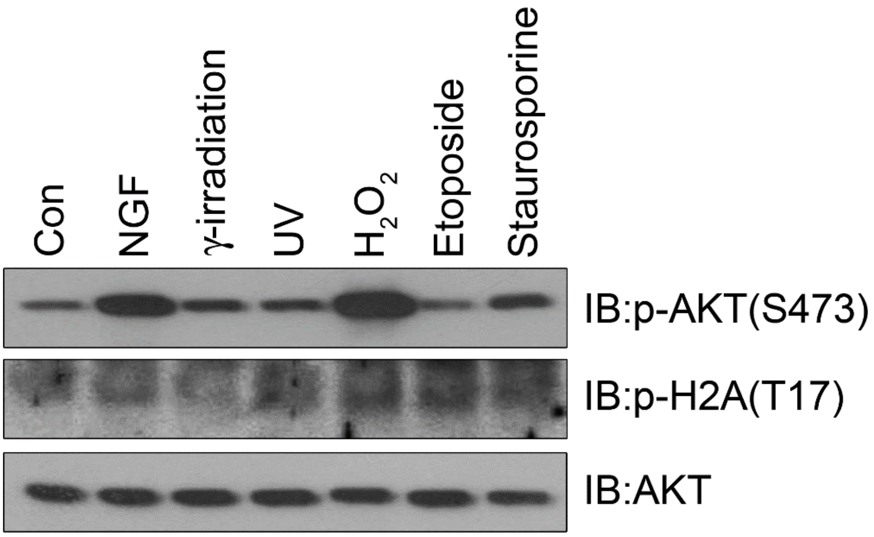

Figure S3 PC12 cells were treated with H_2O_2 in a time-dependent manner for 0.5 to 4.0 h. Active Akt was detected at the 30min time point. The level of pAkt was maintained from 0.5 to 2 h.

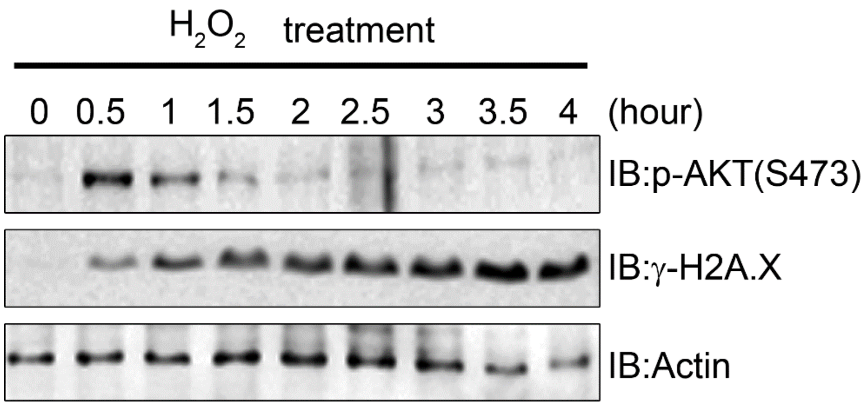

Figure S4 (a) PC12 cells, infected with transfected with sh(short hairpin) RNA-Akt and mock (control) for 24 hours, were exposed to H_2O_2 (1 mM) as indicated time, followed by immunostaining using indicated antibodies and Hoechst. (b) Infection of adenovirus-GFP-Mock and AKT were detected with green fluorescent protein in PC12 cells which are transfected with shRNA-Akt. (c) PC12 cells, transfected with shRNA-Akt and mock (control), were infected with Adenovirus-GFP-Akt and then exposed to H_2O_2 (1 mM) as indicated time, followed by immunostaining using indicated antibodies and Hoechst. (d) Neuron cells exposed to H_2O_2 were stained with Hoechst33342. Apoptotic cells were detected by TUNEL assay (green).

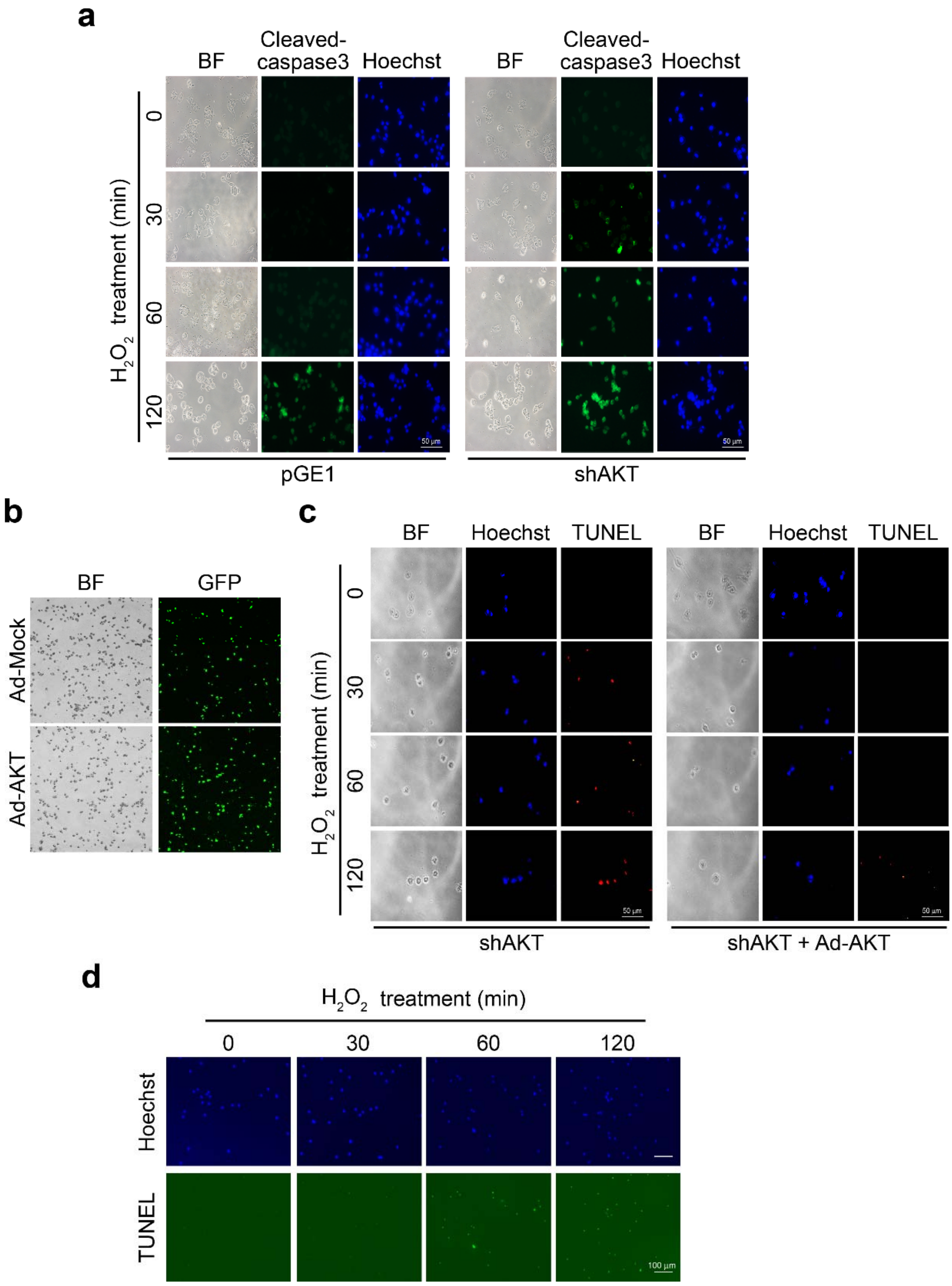

Figure S5 PC12 cells expressing silencing of Sirt2 were detected with green fluorescent protein in cells.

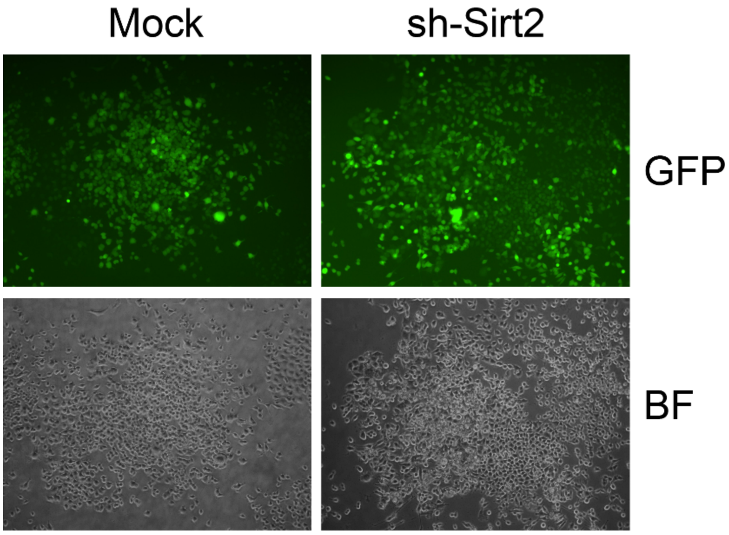

Supplement: Supplementary Information [file srep21857-s1.pdf]
